# Implementation of a cost-effective strategy to prevent neonatal early-onset group B haemolytic streptococcus disease in the Netherlands

**DOI:** 10.1186/1471-2393-13-155

**Published:** 2013-07-30

**Authors:** Diny GE Kolkman, Marlies EB Rijnders, Maurice GAJ Wouters, M Elske van den Akker-van Marle, CPB Kitty van der Ploeg, Christianne JM de Groot, Margot AH Fleuren

**Affiliations:** 1 Department of Child Health, TNO, PO Box 2215, 2301 CE Leiden, The Netherlands; 2 Department of Obstetrics and Gynaecology, VUmc, VU University Medical Centre, PO Box 7057 1007 MB Amsterdam, The Netherlands; 3 Department of Medical decision making, Leiden University Medical Center, PO Box 9600, 2300 RC Leiden, The Netherlands; 4 Department of Life Style, TNO, PO Box 2215, 2301 CE Leiden, The Netherlands

**Keywords:** Early-onset Group B streptococcus, Prevention, Dutch maternity care, Implementation, Guidelines

## Abstract

**Background:**

Early-onset Group B haemolytic streptococcus infection (EOGBS) is an important cause of neonatal morbidity and mortality in the first week of life. Primary prevention of EOGBS is possible with intra-partum antibiotic prophylaxis (IAP.) Different prevention strategies are used internationally based on identifying pregnant women at risk, either by screening for GBS colonisation and/or by identifying risk factors for EOGBS in pregnancy or labour. A theoretical cost-effectiveness study has shown that a strategy with IAP based on five risk factors (risk-based strategy) or based on a positive screening test in combination with one or more risk factors (combination strategy) was the most cost-effective approach in the Netherlands. IAP for all pregnant women with a positive culture in pregnancy (screening strategy) and treatment in line with the current Dutch guideline (IAP after establishing a positive culture in case of pre-labour rupture of membranes or preterm birth and immediate IAP in case of intra-partum fever, previous sibling with EOGBS or GBS bacteriuria), were not cost-effective. Cost-effectiveness was based on the assumption of 100% adherence to each strategy. However, adherence in daily practice will be lower and therefore have an effect on cost-effectiveness.

**Method/Design:**

The aims are to: a.) implement the current Dutch guideline, the risk-based strategy and the combination strategy in three pilot regions and b.) study the effects of these strategies in daily practice. Regions where all the care providers in maternity care implement the allocated strategy will be randomised. Before the introduction of the strategy, there will be a pre-test (use of the current guideline) involving 105 pregnant women per region. This will be followed by a post-test (use of the allocated strategy) involving 315 women per region. The outcome measures are: 1.) adherence to the specific prevention strategy and the determinants of adherence among care providers and pregnant women, 2.) outcomes in pregnant women and their babies and 3.) the costs of each strategy in relation to the effects.

**Discussion:**

This study will provide recommendations for the implementation of the most cost-effective prevention strategy for EOGBS in the Netherlands on the basis of feasibility in daily practice.

**Trial registration:**

Dutch Trial Register, NTR3965

## Background

Vertical transmission of the group B haemolytic streptococcus (GBS) during delivery is the main cause of neonatal infection in the first week of life and it leads to early-onset Group B haemolytic streptococcus disease (EOGBS). EOGBS is an important cause of perinatal mortality, serious illness and long-term effects for the baby [1,2]. The mortality rate for children with EOGBS varies from 9.0 to 10.3% [3-5].

Estimates of the incidence of EOGBS vary between 0.23 and 1.22 per 1000 live births in several European countries and the USA [6-14]. The estimated incidence of EOGBS in the Netherlands is based on proven (0.36 per 1000 live births) [13] and probable EOGBS cases and is 1.9 per 1000 live births [1].

Because of the severe consequences, the importance of the prevention and mitigation of EOGBS is widely recognised. Primary prevention of EOGBS is possible with intra-partum antibiotic prophylaxis (IAP) for pregnant women who are GBS carriers. Different prevention strategies are used internationally based on identifying mothers at risk, either by screening for maternal colonisation with GBS during pregnancy or by identifying risk factors for EOGBS in pregnancy or during labour. Established risk factors include the pre-labour rupture of membranes (> 18 hours), intra-partum fever (≥38.0°C), preterm birth (<37 weeks), GBS bacteriuria in current pregnancy and a previous sibling with EOGBS.

However, none of the preventive EOGBS strategies in place at present result in the complete prevention of EOGBS, either because the strategy is not fully effective in itself, or because it has drawbacks which may result in non-adherence by both care providers and pregnant women.

1. In the risk-based strategy, all women with one or more of the established risk factors for EOGBS receive IAP. Care providers are clear about how to apply this strategy. The effect of the strategy is limited because over 40% of babies who develop EOGBS are born to mothers without a risk factor during pregnancy or labour [13].

2. In the screening strategy, a culture is taken at 35–37 weeks of gestation, and all GBS-colonised women receive IAP. As with the risk-based strategy, care providers understand clearly how to apply this strategy, which is affected by two problems: 1. the failure of the laboratory to detect GBS in pregnant women accurately accounts for a consistent proportion of EOGBS cases [6,15-18] and 2. the large numbers of women receiving IAP and the possible negative side-effects such as antibiotic resistance [6].

3. In the combination strategy, a culture is taken at 35–37 weeks of gestation and women with both GBS colonisation and one or more of the established risk factors for EOGBS receive IAP. This strategy leads to the most accurate use of IAP and therefore results in the lowest rate of negative side-effects. However, persistent problems are that laboratories fail to detect GBS in pregnant women accurately [6,15-18] and over 40% of babies who develop EOGBS are born to mothers without a risk factor during pregnancy or labour [13]. Furthermore, adherence to this strategy may be affected by awareness among care providers and pregnant women of GBS colonisation and this could lead to an increase in IAP, even in the absence of risk factors.

4. The current Dutch guideline, which was issued in 1998 [19], prescribes IAP for women with intra-partum fever, a previous sibling with EOGBS or GBS bacteriuria during current pregnancy. Cultures are taken in cases of pre-labour membrane rupture or preterm birth and IAP is given if GBS colonisation is established. One of the problems with the Dutch guideline is that culture results take up to 72 hours to deliver and cultures are therefore often not yet available when labour starts. IAP in response to a risk factor is therefore predominantly based on clinical observation. The problems mentioned above – the inaccurate detection of GBS in the combination strategy [6,15-18] and the fact that over 40% of babies who develop EOGBS are not born to mothers with risk factors [6]– are also applicable to the Dutch guideline. 

The effect of the introduction of the Dutch guideline on the prevention of EOGBS was first evaluated in 2007 [13]. Trijbels et al. reported that the incidence of neonatal GBS disease fell hardly at all after the introduction of the guideline and recommended a change to the national guideline. In 2005, Van den Akker et al. compared the cost-effectiveness of the Dutch guideline with the risk-based strategy, the screening strategy and the combination strategy in a theoretical model [20]. This analysis took into account the unique maternity care system in the Netherlands, which involves a 31% home birth rate and a stratified care model with different professional care providers at different risk levels. It found that, assuming 100% adherence by care providers and clients, the combination strategy and the risk-based strategy would have the most favourable cost-effectiveness ratios. The screening strategy was the most effective, but the least cost-effective strategy, whereas the Dutch guideline had less effect but involved even higher costs.

However, the actual impact of any of these four strategies will be the product of efficacy (in other words, to what extent can the strategy prevent EOGBS?) and the level of implementation (the extent to which the strategy is followed by all care providers and all pregnant women). Full adherence is unlikely in daily practice. There are many reasons why care providers cannot always observe guidelines: time constraints, lack of patient cooperation, lack of self-efficacy, difficult logistical procedures or even a lack of clear procedures in the guideline, to name just some examples [21-24].

In 2012, there was a study analysing the determinants of the uptake of the four GBS prevention strategies in the Netherlands mentioned here. The study looked at care providers and pregnant women by means of focus group interviews and a subsequent questionnaire study. As well as identifying the determinants associated with each prevention strategy, the study found room for improvement in adherence to the current Dutch guideline, especially in the assessment of clinical risk factors and the process of culture taking. Furthermore, there was not enough support for the introduction of the screening strategy. This strategy also turned out not to be cost-effective in the theoretical model and so it was decided not to introduce this strategy in the Netherlands.

The aim of our study is to implement the current Dutch guideline, the risk-based strategy and combination strategy, and to study the effects in daily practice.

Our results will support recommendations for the national implementation of the most cost-effective EOGBS prevention strategy on the basis of feasibility in daily practice.

The research questions are:

a. What are the levels of adherence to each prevention strategy and the determinants of adherence among care providers and pregnant women?

b. What are the outcomes in pregnant women and their babies?

c. What are the costs of each prevention strategy and how do they relate to the effects?

## Methods/Design

### Innovation framework

In the present study, we use a framework based on several theories that have proven useful in the past for the introduction and evaluation of innovations – such as guidelines – in a wide range of settings in Dutch health care [25-30].

**Figure 1:**
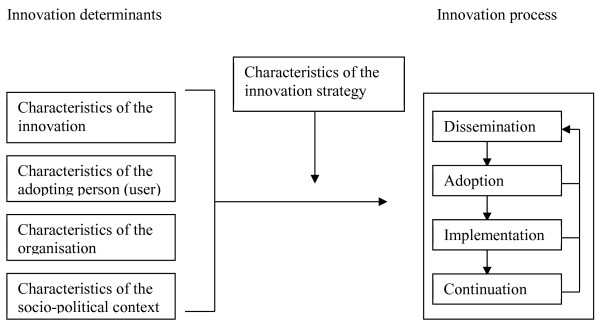
**Framework representing the innovation process and related categories of determinants **[23]**.**

Figure 1 shows the four main stages in innovation processes. In the dissemination stage, the innovation should reach every professional. In the adoption stage, the professional develops positive or negative intentions about using the innovation. In the implementation stage, the professional tries to use the innovation in daily practice and finds out what working with the innovation actually means. In the final stage, the continuation stage, working with the innovation either becomes routine practice or not.

These four main stages in innovation processes can be thought of as success or failure points at which the desired change may, or may not, occur. The transition from one stage to the next can be affected, positively or negatively, by various determinants (see Figure 1). Determinants can be broken down depending on their association with:

1. The innovation (determinants such as complexity, relative advantage, compatibility),

2. The adopting person (determinants such as outcome expectations, self-efficacy, perceived patient cooperation),

3. The organisation (determinants such as staff turnover, financial resources, available time),

4. The socio-political context (determinants such as legislation) [23,24].

A detailed understanding of determinants helps to design an innovation strategy that can achieve real change [23,31-33]. A determinant analysis was performed in 2012 (see Background), resulting in a set of determinants that the present study will anticipate (see Activity 1).

#### The Dutch maternity care system

The Dutch EOGBS guideline targets obstetricians, midwives and paediatricians working in the Dutch maternity care system. This system is a stratified care model with different professional care providers at different risk levels. Midwives working in primary care are the main group of caregivers in low-risk pregnancies. Obstetricians and midwives working in hospitals take care of medium- or high-risk pregnancies and births. Care pathways are organised in Obstetric Collaboration Groups (OCGs). An OCG is organised around a hospital and consists of midwives (with independent practices and/or hospital-based), obstetricians and paediatricians. The OCGs make agreements about the regional organisation of maternity care and interdisciplinary collaboration.

### Overview of project

This project comprises two main activities: 1). the development and application of innovation strategies to enhance the uptake of the three prevention strategies in three study regions and 2). the study of the effects of the implementation. Before the introduction of the three prevention strategies in August 2013, a pre-test will be conducted over a period of three months (March-May 2013). Then, over a period of two months, implementation activities will take place (June-July 2013). To assess the effect of the implementation, a post-test will be performed over a period of six months (August 2013-January 2014).

### Activity 1: Development and application of innovation strategies

#### Participants

Three OCG regions will be recruited. Each OCG consists of one hospital working with three to five midwifery practices. The EOGBS prevention strategy to be implemented – the Dutch guideline, the risk-based strategy or the combination strategy – will be randomly allocated to each region.

All the respondents who participated in the previously performed determinant analysis (see Background) will be asked if their OCG is interested in participating in the study. All the care providers who respond positively will be approached. The chairpersons of the OCGs in the region will then be contacted. If participating members of the OCG express interest, the region will be informed about the study protocol in an OCG meeting. Inclusion criteria for participation are:

a. All OCG members have to participate in the study as the prevention strategy will be implemented in the entire region. If, for example, one member in the OCG does not initially support the allocated prevention strategy, this will have a direct effect on implementation and the cost-effectiveness.

b. The OCG must consist of one hospital and three to five midwifery practices in primary care in the vicinity. The number of care providers participating will be approximately ten midwives in primary care, five obstetricians, eight to ten hospital-based midwives and five paediatricians.

c. The OCG must consider collaboration between the professionals to be good.

#### Innovation strategies

##### 1. Providing information (enhancing dissemination and adoption)

To enhance awareness of the allocated prevention strategy, all the care providers will receive a personal letter and the project will be discussed at several regular meetings of the OCG. Furthermore, a patient information brochure about the prevention strategy will be developed for distribution by all care providers to their clients.

##### 2. Recruitment of coordinators (enhancing adoption, implementation and continuation)

Two implementation coordinators will be assigned to each participating OCG: a midwife and an obstetrician who introduce and guide, coordinate and monitor the implementation process for the allocated prevention strategy. The coordinators will receive coaching throughout the project from an implementation expert and the project group.

##### 3. Training of professional care providers (enhancing adoption and implementation)

Before the introduction of the prevention strategy in August 2013, all care providers will receive training with the aim of optimising the implementation process. The training will be based on the determinants identified in the determinant analysis performed in the past. The training consists of three components:

a. General information will be provided about the theoretical background to EOGBS and its prevention. There will be a particular focus on correct culture-taking since considerable variation was found among care providers in the determinant analysis. The aim is to train all care providers to the same level.

b. The allocated prevention strategy, and the study protocol, will be discussed in detail. Potential barriers to adherence will be discussed and particular attention will be paid to logistics. The aim is to clarify and standardise the allocated strategy for the EOGBS prevention.

c. There will be a particular focus on counselling for pregnant women, emphasising clear and unambiguous information about the screening procedure, the consequences, the administration of IAP and shared decision-making about treatment options.

### Activity 2: Study of the effects of the implementation of each prevention strategy

#### Participants and study design

Before the actual introduction of the three prevention strategies in August 2013, a pre-test will be conducted over a period of three months to assess adherence to the current Dutch guideline. To determine the effects of the implementation of the three strategies, a post-test will be performed over a period of six months. During both the pre-test and post-test, all midwives and obstetricians will prospectively register all pregnant women from 30 weeks of gestational age onwards. All pregnant women will receive information about the EOGBS prevention strategy in their region and permission is acquired on the basis of opting out [34]. Women who decline to participate will be treated in accordance with the current Dutch guideline. However, pregnant women will be explicitly asked for permission to take cultures (see outcome measures) and for IAP in line with the usual professional standards. Therefore written informed consent will be obtained from all pregnant women.

#### Sample size

To determine adherence to each strategy with sufficient precision, 315 women are needed (in the case of 70% adherence, the 95% confidence interval will be 65-75%) since this number will ensure statistical significance at a variation in adherence of 10% between strategies (alpha = 0.05, power = 80%).

The pre-test must include 105 pregnant women per region: 315 in total for the three regions. The post-test must include 315 pregnant women per region: 945 in total for the three regions.

#### Adherence and determinants of adherence

##### Adherence

Throughout the study period, adherence to the key components of each prevention strategy will be measured for all care providers on the basis of the medical records of all the pregnant women. During the pre-test, adherence to the current Dutch guideline will be measured, and adherence to the allocated prevention strategy will be measured during the post-test. The key components are: assessment of the clinical risk factors for EOGBS, culture-taking during pregnancy and during labour, IAP during labour and treatment of the baby. In this way, the level of adherence will be measured as the proportion of all prescribed activities the professional has actually applied: “completeness of use”. The medical record search will be performed using standardised registration forms. Only the combination strategy requires a recto-vaginal culture to be taken from pregnant women between 35 and 37 weeks by their care provider. In all prevention strategies the care provider decides whether IAP is necessary or where there is a contra-indication for antibiotic prophylaxis.

##### Determinants

At the end of the pre-test (and therefore prior to the implementation of the innovation strategies) and at the end of the post-test, all care providers will receive a questionnaire about the – anticipated – determinants of adherence to the allocated prevention strategy. The Measuring Instrument for Determinants of Innovations will be used, which consists of 29 generic determinants that predict the actual use of innovations [24].

#### Outcomes in pregnant women and their babies

Throughout the study period, in the 35th week of pregnancy, all women will receive a questionnaire covering background characteristics, their worries in general and specifically their worries about EOGBS.

In the first week after birth, all women will receive a questionnaire covering actual care received and their satisfaction with received care during pregnancy, labour and the post-partum period.

GBS colonisation of the baby will be used as a proxy measurement for EOGBS. Cultures will be taken from 315 babies during the pre-test and from 945 babies during the post-test.

Clinical outcomes will also be obtained from the medical records: the established risk factors for EOGBS, outcomes of culture-taking during pregnancy and labour, IAP during labour, GBS colonisation of the baby, antibiotic treatment for the baby, length of hospital stay and level of hospital care (standard, medium, high). Standardised registration forms will be used to collect the medical data. Follow-up of the women and children will be one week after delivery.

#### Costs of the prevention strategies in relation to the effects

The decision analysis model of the 2005 study [20] comparing societal costs and the effects of different prevention strategies will be updated and validated using the empirical data from the present study. The costs of implementing the different strategies will also be incorporated. Sensitivity analyses will vary crucial model parameters to assess their influence on the cost-effectiveness ratio.

#### Analysis

Data will be analysed according to the intention to treat principle. OCGs will be analysed on the basis of the allocated strategy, regardless of whether the strategy is fully implemented in the OCG. Base characteristics will be described. The differences in the outcome percentages for the pre-test and the post-test will be calculated and included in the cost-effectiveness analysis. Descriptive and regression techniques will be used to evaluate the effect of the implementation.

### Ethical consideration

This study is approved by the National Central Committee on Research involving Human Subjects (CCMO NL 41673.058.12) and by the ethics committee of the Leiden University Medical Centre (ref. no P12.184). The trial is registered in the Dutch Trial Register NTR 3965, http://www.trialregister.nl/trialreg/admin/rctview.asp?TC=3965).

## Discussion

The effective prevention of EOGBS will be the product of the efficacy of the applied prevention strategy (to what extent can the strategy in itself prevent EOGBS) and the level of implementation (to what extent is the strategy adhered to by all care providers and all pregnant women). Since improved adherence may enhance EOGBS prevention, our study focuses on determinants that affect adherence to three prevention strategies in daily practice. The empirical data about adherence will be used to assess the cost-effectiveness of each strategy in daily practice.

Although the results will primarily be used for the national implementation of the most cost-effective strategy in the Netherlands, the results could be of interest to health-care professionals, policymakers and insurance companies in other countries. Regardless of the prevention strategy used, we expect that other countries will also have problems with actual adherence. Our determinant analysis found a variation in adherence to several recommendations of the guideline, especially in the assessment of clinical risk factors and culture-taking procedures. In the United Kingdom also, considerable variation in the management of EOGBS risk prevention was recently reported, including the application of risk factors and the choice of antibiotics [35].

Our study has some limitations. Due to the low prevalence of EOGBS, a clinical effect study for the detection of a statistically significant difference in the prevalence of EOGBS is not feasible. Our study will use the incidence of neonatal colonisation as a proxy measure for implementation. A second limitation is that the results may be affected by regional variations. To overcome this problem we will include regions with minimal differences in the organisation of maternity care (OCGs) and population characteristics.

We expect the results of our study to contribute significantly to the national and international re-evaluation of the best strategy for the prevention of EOGBS.

## Abbreviations

EOGBS: Early-onset Group B haemolytic streptococcus; IAP: Intra-partum antibiotic prophylaxis; GBS: Group B haemolytic streptococcus; NTR: Nederlands Trial Register/ Dutch trial register; OCG: Obstetric Collaboration Group; CCMO: Central Committee on Research involving Human Subjects.

## Competing interests

This study received funding from the Netherlands Organisation for Health Research and Development (ZonMW), grant number: 200320008.

The authors declare that they have no competing interests.

## Authors’ contributions

MEBR, MGAJW, MAHF, MEvdAvM and CPBvdP were involved in conception and design of the study. DGEK, MAHF, MEBR, MGAJW, MEvdAvM, CPBvdP and CMGdG drafted the manuscript. All the authors listed are members of the ‘GBS study group’. All the authors read and approved the final manuscript.

## Pre-publication history

The pre-publication history for this paper can be accessed here:

http://www.biomedcentral.com/1471-2393/13/155/prepub
